# Ultrasound-guided thermal ablation versus laparoscopic surgery for focal nodular hyperplasia of the liver: A retrospective controlled study

**DOI:** 10.3389/fonc.2022.932889

**Published:** 2022-08-04

**Authors:** Dan-ling Zhang, Sheng Chen, Yu-cheng Lin, Wenxin Ye, Kai Li, Song-song Wu

**Affiliations:** ^1^ Department of Ultrasonography, Fuzhou No.7 Hospital, Shengli Clinical College of Fujian Medical University, Fuzhou, China; ^2^ Department of Ultrasonography, Fujian Provincial Hospital, Shengli Clinical College of Fujian Medical University, Fuzhou, China; ^3^ Department of Ultrasonography, Affiliated Fuzhou First Hospital of Fujian Medical, Shengli Clinical College of Fujian Medical University, Fuzhou, China; ^4^ Department of Ultrasonography, Sanming Second Hospital, Shengli Clinical college of Fujian Medical University, Sanming, China; ^5^ Department of Ultrasound, Guangdong Key Laboratory of Liver Disease Research, The Third Affiliated Hospital of Sun Yat-sen University, Guangzhou, China

**Keywords:** thermal ablation (Ab), surgery, radiofrequency ablation, microwave ablation, focal nodular hyperplasia

## Abstract

**Purpose:**

This study aims to evaluate the value of the clinical application of ultrasound-guided percutaneous thermal ablation in focal nodular hyperplasia (FNH) by comparing its safety, effectiveness, and patient experience to surgery in the treatment of hepatic FNH ≤5 cm.

**Method:**

This retrospective study enrolled 82 patients with hepatic FNH having a maximum diameter of ≤5 cm, confirmed by postoperative pathologic diagnosis or needle biopsy, who underwent thermal ablation or surgery between January 2019 and September 2021. Postoperative efficacy, surgical trauma (operation time, intraoperative bleeding volume, liver function, and lost volume of normal liver tissue), postoperative complications (postoperative infection, pleural effusion, and liver dysfunction), patient experience (degree and time of postoperative pain, postoperative fasting time, indwelling thoracic chest drain, and scar size), and economic indices (postoperative hospitalization and total charges) were compared between both groups.

**Result:**

No significant difference existed in postoperative efficacy between both groups (*p* > 0.05). No recurrent or new lesions were observed during the 6-month follow-up in both groups. However, significant differences were observed in operation time, intraoperative bleeding volume, and lost volume of normal liver tissue (*p* < 0.05), with significantly less trauma in the thermal ablation group. No statistically significant differences in ALT, AST, and Hb existed between both groups (*p* > 0.05); however, albumin was higher in the ablation group compared to the surgery group (38.21 ± 3.32 vs. 34.84 ± 3.71 g/L, *p* < 0.05), and WBC were lower in the ablation group (11.91 ± 3.37 vs. 13.94 ± 3.65/L, *p* < 0.05). The incidence of postoperative complications in the ablation group was significantly lower than that in the surgery group (*p* < 0.05). Patient experiences were significantly better than in the surgical group (*p* < 0.05), with economic indicators being significantly less in the ablation group (*p* < 0.05).

**Conclusion:**

Ultrasound-guided percutaneous thermal ablation can treat hepatic FNH ≤5 cm with similar clinical efficacy as surgery and is an economical, safe, and minimally invasive treatment method worthy of recommendation.

## Introduction

Hepatic focal nodular hyperplasia (FNH), a proliferative lesion in which the liver parenchyma proliferates and is separated into nodules by stellate fibrous scars (WHO criteria) (1), has an incidence of 0.6%–3% in the general population (2). Its current pathogenesis is also unclear. It is widely believed that its pathogenesis is due to neoplastic hyperplasia (regenerative nodules) or vasodilation caused by the body’s adaptive response to tissue damage (3). Therefore, this lesion is a benign space-occupying lesion with nonnatural growth and no malignant lesion; moreover, the majority have no risk of bleeding.

Since most FNH patients have normal laboratory values with no special clinical manifestations, diagnosis is mainly based on imaging examination. In recent years, with improvement in imaging techniques and deepened understanding of FNH by clinicians, its diagnostic level has significantly improved. ACG clinical practice guidelines on the management of focal liver lesions (4) propose that a conservative approach should be taken when managing FNH. However, further evaluation of symptomatic lesions in which a diagnosis of FNH cannot be firmly established is recommended. Relevant scholars have also made in-depth research on the management of FNH diagnosis and treatment. Zarfati (5) summarized and analyzed the management experience of 50 FNH pediatric patients to propose that watchful waiting is a safe initial approach to pediatric FNH management in patients with no major symptoms or complications. Surgery should be reserved for patients with diagnostic doubt, persistent symptoms, and/or biological or significant anatomical abnormalities. Jung et al. (6) analyzed the surgical indications of 48 adult FNH cases in a single center and showed that FNH can be diagnosed by imaging studies, but surgical treatment may be considered in cases of diagnostic uncertainty or persistent symptoms. So, although the vast majority of FNH only needs to observed, a few cases of diagnostic uncertainty or persistent symptoms still need surgical treatment. At present, the main treatment methods for FNH are surgery and transarterial embolization (TAE). Generally, open or laparoscopic FNH resection is considered the preferred treatment (7). However, liver surgery can cause surgical trauma, such as significant bleeding, a high risk of postoperative complications, and an obvious scar (8). Although TAE, as a minimally invasive treatment, can reduce lesion volume and control pain preoperatively, it also has the risk of residual and increased radiation exposure (9). Therefore, providing treatment that is more safe, effective, and economical, with minimal trauma for such FNH patients who need active treatment should be considered. In recent years, thermal ablation, as the leading edge of minimally invasive treatment, has been widely used in the treatment and research of tumors of the liver, lung, and kidney, and has achieved good results (10–12). Thermal ablation and surgical resection have been listed as radical methods for treating small liver cancer internationally, including by the American Association of Liver Diseases (AASLD), the European Association of Liver Diseases (BCLC), Asia Pacific Association for the Study of the Liver (APASL), and the Chinese guidelines for the diagnosis and treatment of primary liver cancer (13). Currently, there are also some reports on thermal ablation of hepatic FNH, all of which suggest a good curative effect. Yaz (14) reported successfully treating FNH in a 9-year-old child using a microwave. Yu et al. (15) confirmed the safety and effectiveness of FNH thermal ablation in a multicenter study. Regarding the effect of thermal ablation in FNH treatment, there has been no comparative study on the efficacy and risk-benefit balance between thermal ablation and surgery in current domestic and foreign studies. In this paper, the clinical data of patients with FNH confirmed by pathology were reviewed retrospectively to evaluate the value of the clinical application of thermal ablation compared to surgical treatment in terms of clinical safety, efficacy, patient experience, and economic effect, to provide a basis for selecting and formulating the best treatment plan for FNH patients requiring aggressive treatment.

## Materials and methods

### General clinical data

This study was approved by the ethical and scientific review board of Fujian Provincial Hospital. Medical records of 103 patients with hepatic FNH with a maximum diameter of ≤5 cm confirmed by postoperative pathologic diagnosis or needle biopsy, who underwent thermal ablation (*n* = 53) or surgery (*n* = 50) in Fujian Provincial Hospital and the Third Affiliated Hospital of Guangzhou Sun Yat-Sen University between January 2019 and September 2021 were reviewed. “Lost to follow-up” was defined as failure to obtain complete follow-up data. Patients were included based on the inclusion and exclusion criteria, and patients lost to follow-up were excluded. A total of 82 patients (79.6%, 82 of 103) met the inclusion criteria, of which 39 (73.6%, 39 of 53) with 49 lesions (three multiple lesions, 36 single lesions) underwent thermal ablation (thermal ablation group) and 43 (86%, 43 of 50) with 50 lesions (three multiple lesions, 37 single lesions) underwent surgery (surgery group).

The inclusion criteria were as follows: (1) patients with FNH confirmed by postoperative pathology or biopsy; (2) FNH lesion diameter ≤5 cm; (3) normal preoperative coagulation; and (4) clear preoperative lesion and number imaging.

The exclusion criteria were as follows: (1) patients unwilling to undergo surgery or thermal ablation; (2) FNH lesion diameter >5 cm; (3) patients with other important organ diseases requiring combined operation; and (4) incomplete follow-up data.

### US-guided thermal ablation

**Figure 1:**
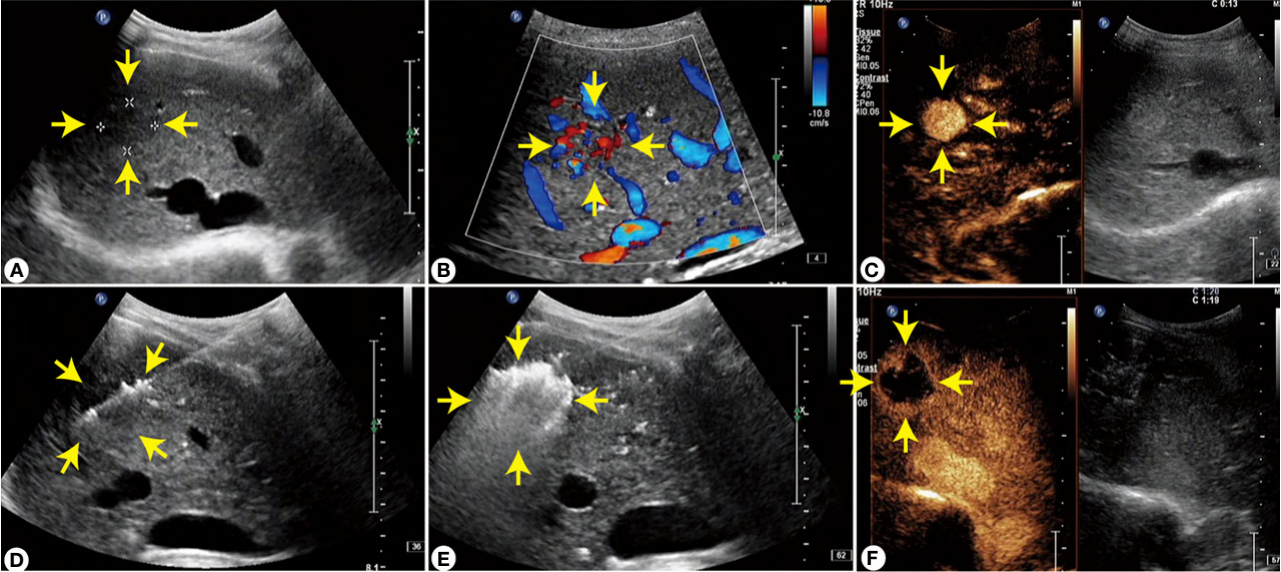
The whole process of ultrasound-guided abltion of S8 segment of FNH lesions. **(A)** Two-dimenstional ultrasound imagine of FNH lesions. **(B)** Color Doppler flow magine of FNH lesions. **(C)** FNH contrast-enhanced ultrasound images in arterial phase Cover. **(D)** FNH lesion ablation needle enters the proper position to start ablation. **(E)** The lesion is ablated until fully covered by hyperechoic echoes. **(F)** Contrast-enhanced ultrasound 15 minutes after ablation indicates that the lesion has no enhancement.

Thermal ablation was performed using a Viva RFA system (hostmode VRS01, STARMed, South Korea) with a disposable monopolar RF ablation needle electrode (model 18-07s07F; working electrode length, 7 mm) or a Microwave Ablation Therapy Instrument (model KY-2000, Kangyou, Nanjing) with a disposable microwave ablation needle (model KY-2450A-10; needle length 200 mm). Ultrasonography and contrast-enhanced ultrasonography were performed using the Philips IU22 with C5–2 low-frequency convex array probe to determine the specific location, number, and size of the lesion; also, an RFA or microwave ablation pathway was designed. The patient was placed in a supine position with the upper abdomen fully exposed. Sufentanil at 0.01–0.02 µg/kg and midazolam at 0.02–0.03 mg/kg were intravenously administered for sedation and analgesia, then the skin was disinfected and covered with a sterilized towel. In total, 2% lidocaine was used to locally infiltrate the skin at the anesthesia puncture site of the anterior capsule of the liver. The RFA or microwave ablation needle was inserted into the intrahepatic lesions. The ablative power was set at 100–120 W; the average temperature was controlled at 90°C and starting from the posterior and lower part of the lesion, gradual forward advancement was done needle-by-needle. Tumors with a diameter of <2 cm were ablated using point-one needle ablation, and tumors with a diameter of >2 cm were ablated by multipoint and multineedle ablation. For those clearly diagnosed on imaging, the inactivation range should cover the tumor completely; meanwhile, for those with no obvious diagnosis, the inactivation range should be 0.5–1 cm larger than the outer edge of the tumor (see **Figure 1** for schematic diagram of the ablation range and ablation process). After complete ablation, the ablation needle was slowly withdrawn and the needle track was ablated to stop any bleeding. Finally, color Doppler ultrasonography and contrast-enhanced ultrasonography were performed to confirm the complete inactivation of the intrahepatic lesions. Ablation was considered effective when the contrast agent could not be perfused into the whole tumor. Subsequently, the patient underwent an ultrasound-guided coarse needle biopsy, and ablation was completed after no bleeding was observed. All thermal ablative procedures were performed by the same experienced treatment group.

### Surgical procedure

**Figure 2:**
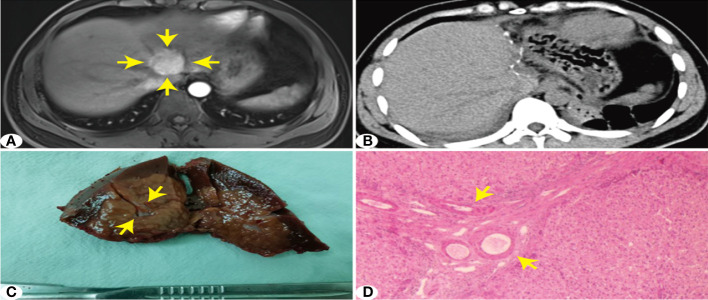
Surgical hepatectomy of S4 segment FNH lesions. **(A)** MRI arterial phase enhanched image. **(B)** Postoperative CT image. **(C)** FNH surgical resection of gross specimens (small spacimens are FNh lesions, and large specimens are the resction range of normal liver tissue. **(D)** Liver FNH microscope image.

After induction of general anesthesia by endotracheal intubation, the patient was placed in a scissor position. Routine disinfection and toweling were conducted. A pneumoperitoneal needle was punctured 1 cm below the umbilicus to establish an artificial pneumoperitoneum, and the pressure was controlled at 10–12 mmHg. According to the preoperative imaging data, the lesion was identified and the laparoscopic instrument was placed. The electrical hook or ultrasonic knife was used to free the perihepatic ligament and confirm the number of tumor sites. The specific surgical method (hepatectomy or anatomic hepatectomy) was determined according to the preoperative imaging examination and intraoperative exploration. An intraoperative ultrasound-guided ultrasonic scalpel combined with an electric hook was used to resect the liver tumor tissue while maintaining hemostasis. After complete wound hemostasis, an abdominal drainage tube was placed. The laparoscopic surgical resection of the left hemi-liver is shown in **Figure 2**.

### Observation indicators and evaluation criteria

Follow-up observation indices were as follows: (1) basic information: patient’s gender and age, tumor diameter, and reasons for the choice of treatment; (2) patient trauma: preoperative and postoperative ALT, AST, albumin, Hb, WBC, operation time, intraoperative blood loss, intraoperative blood transfusion rate, and normal liver tissue loss volume; (3) postoperative complications: postoperative infection, pleural effusion, and severe liver dysfunction; (4) patient experience: postoperative fasting time, indwelling abdominal drainage tubes, scar size, degree and time of postoperative pain, length of hospital stay, and total hospitalization expenses; and (5) efficacy evaluation: lesion disappearance or inactivation and MR- or CT-enhanced follow-up.

The curative effect was evaluated as follows: (1) cure: all target lesions disappeared (negative surgical margin) or were completely inactivated (the entire lesions showed no enhancement on enhanced imaging at all), and there were no new lesions within at least 6 months; (2) improved: the target lesions were partially residual or partially active (residual active lesions <50% of the original lesion volume); and (3) invalid: volume of unresected or residual active lesions were >50%. During the 6-month follow-up period, MR-enhanced assessment was used, and follow-up contents included the presence or absence of new lesions and the residual activity of target lesions.

#### Normal liver tissue loss volume assessment

All patients used CT three-dimensional visualization software (IQQA-LIVER) to perform retrospective three-dimensional reconstruction and calculate the volume of the preoperative liver tumor and nonenhanced area after ablation in the ablation group and the preoperative and postoperative liver and preoperative liver tumor in the surgical group. Normal liver tissue loss volume in the surgery group = preoperative liver volume − postoperative liver volume − preoperative liver tumor volume. Normal liver tissue loss volume in the ablation group = volume of the nonenhanced area after ablation − preoperative liver tumor volume. According to the calculation results, it was divided into two groups of ≥10cm^3^ and <10 cm^3^.

### Statistical analysis

Statistical analysis was performed using SPSS v19.0. The data were reported as mean ± standard deviation. The Student’s *t*-test was used for the statistical comparison of measurements. Fisher’s Chi-square test or *χ* test was used for comparison between count data and categorical variables. *p* < 0.05 was considered statistically significant.

## Results

### Baseline characteristics of the thermal ablation and surgery groups

In total, 82 cases were enrolled in this study, including 43 cases (18 men and 25 women) in the surgical group with 50 lesions and a mean age of 32.05 ± 8.8 years old; 39 cases (17 men, 22 women) formed the thermal ablation group with 49 lesions and mean age of 35 ± 11.5 years. There were no significant differences in gender, age, preoperative maximum tumor diameter, and preoperative laboratory indicators (ALT, AST, albumin, Hb, and WBC) between both groups (*p* > 0.05). The baseline characteristics of each group are summarized in **Table 1**.

**Table 1:** Comparison of baseline characteristics between surgery and thermal ablation.

Variables	Surgery	Thermal ablation	*p*-value	*t*/*χ* ^2^
Male/female	18/25	17/22	0.791	0.071
Age (year)	32.05 ± 8.8	35 ± 11.5	0.195	−1.308
Hb (g/L)	136.00 ± 16.25	134.64 ± 10.54	0.658	0.444
WBC (10^9^/L)	6.75 ± 1.95	7.21 ± 1.34	0.226	−1.221
ALT (U/L)	24.35 ± 23.54	24.05 ± 15.34	0.951	−0.061
AST (U/L)	29.3 ± 34.51	24.05 ± 15.86	0.513	0.657
Albumin (g/L)	45.21 ± 3.87	44.74 ± 2.53	0.468	0.641
Lesions diameter (mm)	32.82 ± 12.14	28.8 ± 9.12	0.061	1.862

ALT, alanine aminotransferase; AST, aspartate aminotransferase; WBC, white blood cells; Hb, hemoglobin. p < 0.05 was considered statistically significant between the two groups.

### Analysis of active treatment factors

Due to high psychological pressure, although FNH has been diagnosed, patients who failed to accept these intrahepatic space-occupying lesions only need treatment observation, accounting for 57.3% (47/82). The nature of the lesions could not be identified by clinical assessment and imaging or the possibility of malignant lesions could not be excluded, accounting for 29.3% (24/82). Among them, 10 had hepatitis cirrhosis and the first diagnosis was liver cancer. Four out of 82 patients had lesion enlargement during follow-up observation (4.9%) and seven out of 82 patients had clinical symptoms (8.53%).

### Comparison of postoperative curative effect in the two groups

The success rate of the two groups was 100%. After repeated re-examination within 6 months, one case in the thermal ablation group had little residue. After secondary ablation and complete ablation, the cure rates were 97.4% and 100%, respectively. There was no significant difference between the two groups (*p* > 0.05) (**Table 2**). No recurrent lesions were found in both groups after 6 months of follow-up.

**Table 2:** Comparison of curative effect between surgery and thermal ablation.

	Completely removed	A small amount of residual	*X* ^2^	*p*-value
Surgery	43	0	0.356	0.332
Thermal ablation	38	1		

p < 0.05 was considered statistically significant between the two groups.

### Comparison and analysis of operation time, intraoperative blood loss, and normal liver tissue loss between the two groups

**Figure 3:**
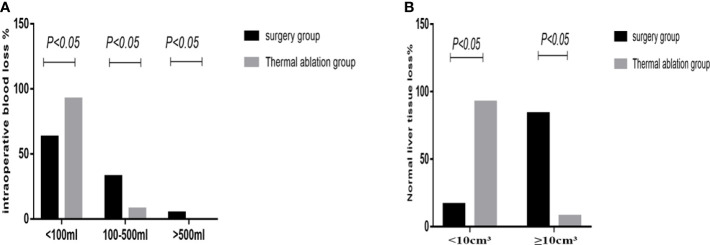
Statistical chart of intraoperative bleeding and loss of normal liver tissue in the two groups. **(A)** Statistical chart of intraoperative bleeding. **(B)** Loss of normal liver tissue statistical chart.

In the surgery group, the average operation time was 137.6 ± 59.7 min; intraoperative blood loss was as follows: bleeding volume <100 ml (62.79%, 27/43), 100–500 ml (32.56%, 14/43), and >500 ml (4.65%, 2/43). Two patients received an intraoperative blood transfusion at a rate of approximately 4.65%; normal liver tissue loss was as follows: volume <10 cm^3^ (16.27%, 7/43) and ≥10 cm^3^ (83.72%, 36/43). In the thermal ablation group, the average operation time was 44.87± 13.3 min; intraoperative blood loss was as follows: bleeding volume <100 ml (92.31%, 36/39) and 100–500 ml (7.69%, 3/39). No intraoperative bleeding >500 ml or blood transfusion occurred; normal liver tissue loss was as follows: volume <10 cm^3^ (92.31%, 36/39) and ≥10 cm^3^ (7.69%, 3/43). There were three patients in the thermal ablation group with intraoperative needle trace hemorrhage, of whom two had liver needle trace hemorrhage and bleeding volume was approximately 100 ml. After needle trace ablation, the bleeding stopped. Another case of hemorrhage occurred in the chest wall. Contrast-enhanced ultrasound (CEUS) can clearly detect chest wall hemorrhage and flow to the right thoracic cavity. The amount of blood loss was approximately 400 ml. Thrombin was injected into the bleeding point to successfully stop the bleeding. No continuous bleeding was observed for 2 days. In the surgery group, 16 patients (37.2%) had intraoperative blood loss >100 ml—two patients had bleeding >500 ml that required intraoperative blood transfusion and seriously increased body trauma. The operation time, blood loss, and normal liver tissue loss in the thermal ablation group were lower than those in the surgery group, and the differences were statistically significant (*p <*0.05) (**Figure 3**).

### Comparison of postoperative laboratory indicators between the two groups

The postoperative laboratory indicators, ALT, AST, albumin, Hb, and WBC, of both groups, were statistically analyzed. There was no significant difference in postoperative ALT, AST, and Hb between the two groups (*p >*0.05). However, postoperative albumin was higher in the thermal ablation group compared to the surgical operation group, while postoperative WBC was lower in the ablation group compared to the surgical operation group; the difference between both groups was statistically significant (*p <*0.05). This comparison is shown in **Table 3**.

**Table 3:** Comparison of ALT, AST, albumin, Hb, and WBC between the two groups (*x* + *s*).

Laboratory indicators	Surgery	Thermal ablation	*t*	*p*-value
AST (U/L)	146.91 ± 138.04	108.13 ± 96.82	1.459	0.149
AST (U/L)	171.65 ± 247.34	121.64+108.6	1.163	0.248
Albumin (g/L)	35.28 ± 3.92	38.21 ± 3.32	−3.634	<0.001
Hb (g/L)	120.71 ± 15.82	122.97 ± 10.08	−0.772	0.443
WBC (10^9^/L)	13.77 ± 3.62	11.91 ± 3.37	2.393	0.019

ALT, alanine aminotransferase; AST, aspartate aminotransferase; WBC, white blood cells; Hb, hemoglobin. p < 0.05 was considered statistically significant between the two groups.

### Comparison of postoperative complications between the two groups

After treatment, 16 and four patients in the surgical and thermal ablation groups had postoperative complications, with an incidence of 37.2% (16/43) and 10.2% (4/39), respectively. The difference was statistically significant (*χ* ^2^: 5.937, *p* = 0.015), and the incidence of complications in the ablation group was much lower than that in the surgery group. In the surgical operation group, there were two cases of infection, one case of severe intracranial and cheek infection, and one case of incision infection, all of which improved after treatment; there were eight cases of postoperative pleural effusion, including two cases of pulmonary infection, six cases of postoperative liver dysfunction (two cases were severe liver dysfunction ALT >500 U/L). In the postoperative ablation group, two cases had pleural effusion and two had severe liver dysfunction (ALT >500 U/L) (**Table 4**).

**Table 4:** Comparison of postoperative complications between the two groups.

	Surgery groups number/rate (%)	Thermal ablation groups number/rate (%)
Pleural effusion	8/18.6	2/5.1
Postoperative infection	2/4.6	0/0
Postoperative liver function damage (ALT > 300 U/L)	6/13.9	2/5.1
Total postoperative complications	16/37.2	4/103

*p < 0.05* was considered statistically significant between the two groups (*χ^2^* = 5.937; *p = 0.015*).

### Comparison of patients’ treatment experience between the two groups

**Figure 4:**
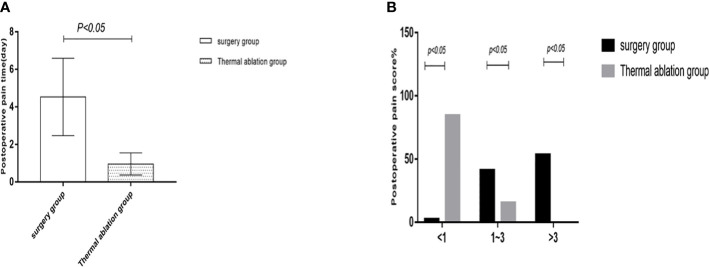
Statistical chart of postoperative pain time and a degree in two groups. **(A)** Statistical chart of postoperative pain time. **(B)** Statistical chart of postoperative pain degree.

**Figure 5:**
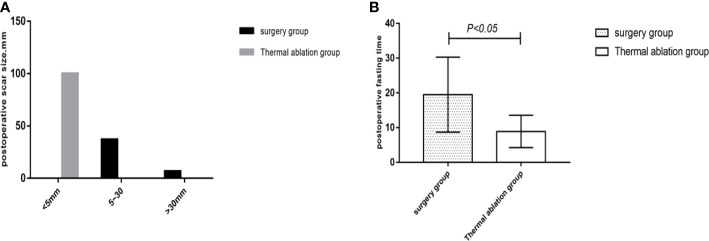
Statistical chart of postoperative scars and fasting time in the two groups. **(A)** Statistical chart of postoperative scars. **(B)** Statistical chart of postoperative fasting time.

Comparison of posttreatment experience between the two groups showed that postoperative degree and time of pain, postoperative fasting time, thoracic and abdominal drainage tube placement, scar size, and postoperative antibiotic use in the thermal ablation group were superior to those in the surgical group, with significant differences between the two groups (*p* < 0.05) (**Tables 5**–**8**; **Figures 4**, **5**).

**Table 5:** Comparison of postoperative fasting time and pain time between the two groups (*x* ± *s*).

	Surgery groups	Thermal ablation group	*t*	*p*-value
Postoperative pain (day)	4.53 ± 2.06	0.96 ± 0.59	11.068	<0.001
Postoperative fasting (hour)	19.53 ± 10.8	8.92 ± 4.67	5.864	<0.001

p < 0.05 was considered statistically significant between the two groups.

**Table 6:** Comparison of postoperative drainage tube emplacement and antibiotic use between the two groups (*x* ± *s*).

	Surgery groups	Thermal ablation group	*χ* ^2^	*p*-value
Drainage tube	42	1	70.412	<0.0001
Emplacement				
No drainage tube	1	38		
emplacement				
antibiotic use	19	1	28.13	<0.0001
No antibiotic use	24	38		

p < 0.05 was considered statistically significant between the two groups.

**Table 7:** Comparison of postoperative pain degree between two groups.

		Postoperative pain score		Total
	<1	I–III	>III	
Surgery groups	1	19	23	43
Thermal ablation groups	33	6	0	39
Totals	34	25	23	82

p < 0.05 was considered statistically significant between the two groups (χ^2^ = 71.025, p < 0.001).

**Table 8:** Comparison of postoperative scar size between two groups.

	Postoperative scar size			Total
	<5 mm	5–30 mm	>30 mm	
Surgery groups	0	16	27	43
Thermal ablation groups	39	0	0	39
Total	39	16	27	82

p < 0.05 was considered statistically significant between the two groups (χ^2^ = 102.39; p < 0.001).

### Comparison of postoperative hospitalization and total charges between the two groups

The postoperative hospital stay and total hospital expenses of patients in the ablation group were significantly lower than those in the surgery group (7.72 ± 5.71 vs. 5.44 ± 3.45 days; 36,776.23 ± 10,114.39 yuan vs. 21,242.99 ± 2,255.59 yuan), with significant differences between both groups (*p* < 0.05).

## Discussion

FNH is the second most common benign hepatic lesion, with a high age of incidence ranging from 20 to 40 years (2). Related literature (16) reported that its incidence significantly differs between genders, with female patients accounting for about 80%~95%. Moreover, the lesions in female patients are often more typical than those in male patients. These studies performed to date show that 87.8% of the age of incidence ranges from 20 to 40 years, with a slight female preponderance in a male to female ratio of 1:1.22. However, there was no significant gender difference, and the results were similar to those reported.

In our retrospective study, among 24 patients (24/82, 29.3%) with suspected FNH but unclear diagnosis whose diagnoses were confirmed by surgery or ablation, 10 had hepatitis cirrhosis and the first diagnosis was liver cancer. Seven patients (7/82, 8.53%) were actively treated for clinical symptoms such as upper abdominal pain. Due to high psychological pressure, although FNH has been clearly diagnosed on imaging, 47 patients (47/82, 57.3%) who failed to accept this intrahepatic space-occupying lesion only needed treatment observation. They refused diagnosis of an intrahepatic spaceoccupying lesion and strongly desired treatment and 4.9% patients (4/82) with lesion enlargement at follow-up observation underwent thermal ablation or surgical treatment. Patients who cannot accept intrahepatic mass and are strong-willed regarding treatment are the main reasons for FNH treatment. Nonactive treatment of such patients will cause serious psychological problems such as anxiety, which do not favor physical and mental health. Providing a more safe and more effective treatment with less trauma for patients who need active treatment for FNH is worth considering.

In recent years, with development in ultrasound diagnosis and treatment technology, ultrasound-guided thermal ablation has been widely used in the minimally invasive treatment of solid liver tumors and has achieved good therapeutic effects, which have been unanimously recognized by clinicians and patients. The Guidelines for Diagnosis and Treatment of Primary Liver Cancer (2022 Edition) (12) clearly pointed out that thermal ablation is suitable for CNLC stage IA and some stage IB liver cancers (i.e., single tumor, diameter ≤5 cm; or 2~3 tumors, maximum diameter ≤3) without blood vessel, bile duct, and adjacent organ invasion or distant metastasis and Child–Pugh class A/B liver function, which can obtain a radical therapeutic effect. Meanwhile, the application of thermal ablation in the treatment of benign solid tumors is also developing rapidly; however, relevant guidelines and specifications are lacking. In recent years, there have been many reports on FNH thermal ablation treatment at home and abroad. Hedayati et al. (17) reported a female patient with severe upper abdominal pain and pathologically confirmed FNH by liver biopsy. Abdominal pain was significantly relieved after RFA treatment. Yu et al. (15) conducted a multicenter study on FNH thermal ablation to confirm its safety and effectiveness, with little trauma and benefits for patients. In this study, thermal ablation achieved good results, with a single ablation cure rate of 97.4% and a second ablation cure rate of 100%. There was no statistically significant difference compared to the surgical effect, and efficacy was comparable to that of surgery.

In this study, the operation time, anesthesia method, intraoperative blood loss, postoperative laboratory examination, and normal liver tissue loss were compared and analyzed to evaluate trauma from thermal ablation and surgery in the treatment of FNH. The trauma analysis of the two FNH treatment methods showed that thermal ablation was significantly less traumatic than surgery. In this study, the total operation time with thermal ablation for FNH was 44.87 ± 13.3 min, which was significantly lower than the surgical time of 137.6 ± 59.7 min. Shortening the operation time can effectively reduce the risk of bleeding and liver dysfunction. Thermal ablation also showed obvious advantages in anesthesia. In thermal ablation, intravenous sedation and analgesia plus local basic anesthesia were mostly used and could reduce the intake of narcotic drugs and the risk of related damage. Most patients had no obvious pain during the treatment process. In terms of intraoperative blood loss, the amount of intraoperative blood loss in the thermal ablation group was significantly lower than that in the surgical operation group (*p* < 0.05). Both thermal ablation and surgical treatment of FNH can cause transient changes in the body’s laboratory parameters; however, the degree of injury is usually mild and usually returns and normalizes within a week. There was no significant difference in postoperative ALT, AST, and Hb between both groups; however, the albumin content in the postoperative surgical group was significantly lower than that in the thermal ablation group (*p*< 0.05). It showed that compared to thermal ablation, surgery results in greater nutrient loss.

The analysis of the postoperative loss of normal liver tissue between both groups showed that 84% (36/43) of the volume of normal tissue lost was >10 cm^3^ during a surgical operation, while only 7.7% (3/39) was >10 cm^3^ in the thermal ablation group. The postoperative loss of normal liver tissue in the surgical group was significantly greater than that in the thermal ablation group (*p* < 0.05). The volume loss of normal liver tissue mainly correlates with enlarging the ablation range and the method of surgical resection used. For FNH lesions with a definite diagnosis, there is no need to enlarge the ablation range. Meanwhile, FNH is rich in blood supply; the ablation heat well surrounds the entire lesion; hence, it causes little damage to normal liver tissue. In this study, 92.3% (36/39) belonged to this situation. For lesions of unclear nature, especially those in which malignancy could not be excluded, ablation must be carried out in strict accordance with the ablation standard of the malignant tumor (0.5–1 cm beyond the edge of the lesion) to ensure effective inactivation of the lesion (18). In this study, there were three cases of enlargement of the ablation range. However, in surgery, in view of the biological characteristics of benign liver tumors, tumor recurrence and the so-called safe margin are not usually considered in hepatectomy; thus, tumor resection should maximize the preservation of a healthy normal liver and minimize intraoperative blood loss and transfusion (19). Nonanatomic local resection or regular liver segment resection or lobectomy is most commonly used in clinical surgery, while hemi-hepatectomy can also be used for a few large lesions. For patients with FNH that are difficult to differentiate from primary liver cancer (20), surgical exploration should be performed with regular hepatic lobectomy and local resection with a “safe margin” in accordance with the criteria for malignant tumors. For the central type and small tumors less than 5 cm located in segments I and VIII, local resection should still be selected to avoid loss of a large amount of liver tissue or serious surgical complications due to small benign tumors. In this study, the volume of normal liver tissue injury >10 cm^3^ was about 83.7% (36/43), and only seven cases of local liver tumor excision had the least normal liver tissue injury. Although the surgical operation and thermal ablation only need to resect the lesion, due to the complexity of the surgical operation, it is difficult to only resect the lesion. Thermal ablation can also follow and can protect normal liver tissue and ensure stable normal digestive function. In conclusion, thermal ablation is superior to surgery in terms of anesthesia, operation time, intraoperative bleeding, and preservation of normal liver tissue with less surgical trauma.

In this study, common complications, such as postoperative infection, pleural effusion, and postoperative severe liver damage, caused by thermal ablation and surgical treatment for FNH were compared and analyzed to evaluate the occurrence of postoperative complications in both groups. This study showed that complications of thermal ablation for FNH were less than those of surgery. There was a significant difference between both groups. In this study, there were four cases (4/39, 10.2%) with complications such as severe liver dysfunction and pleural effusion in the thermal ablation group, including two cases of postoperative pleural effusion and two cases of severe liver dysfunction with transaminase >500 U/L. Both of the patients with severe liver dysfunction had multiple lesions, five lesions in one case and three lesions in the other, which were considered to be caused by ablation of multiple lesions. After treatment for liver preservation, the patients returned to normalcy approximately 2 weeks later. In two cases who had pleural effusion, one was caused by intraoperative chest wall hemorrhage, while the other was a very small amount on the right diaphragm, with no special treatment given; the 1-month follow-up examination was self-absorbed. In the surgical group, there were 16 cases (16/43, 37.2%) of postoperative complications, including eight cases of pleural effusion, two cases of postoperative infection, and six cases of severe liver dysfunction. Among the eight cases of pleural effusion, two were complicated by pulmonary infection, which mainly correlated with long postoperative time in bed. Severe liver dysfunction occurred in six patients postoperatively, which may correlate with operation time and multiple lesions (all six patients had operation time >180 min; two patients had more than three lesions). Among the two infected patients, one suffered incision infections and was cured by drug treatment, while the other patient had incisional and extra-abdominal cavity infection 5 days after the operation, which occurred in the intracranial and buccal cavity, causing speech impediment and motor dysfunction. It improved after anti-infection therapy and rehabilitative exercise for 3–5 months. The inducement of infection may be related to the patient’s long operation time (approximately 240 min) and large intraoperative blood loss (approximately 500 ml), which may cause greater damage to the body and result in significantly weakened resistance. In conclusion, compared to surgical treatment, thermal ablation of FNH has less intraoperative trauma, resulting in fewer postoperative complications than surgical treatment, and the overall incidence of complications is better than that of surgical treatment. This study analyzed the physical and psychological comfort of patients in the treatment of the two groups by comparing postoperative fasting time, indwelling time of abdominal drainage tubes, scar size, degree and time of postoperative pain, length of hospital stay, and total hospitalization expenses. The results showed that the physical and psychological comfort of patients treated with FNH thermal ablation was significantly better than that of patients treated with surgery. After thermal ablation for FNH, there was no need for a drainage tube (only one case was due to pleural effusion drainage), and only 4–8 h of fasting was required under local anesthesia and sedation. The scar was <5 mm, and there was less postoperative hospitalization time and total treatment costs, all of which were beneficial to patients. Small trauma and a comfortable treatment experience can help patients recover quickly. In conclusion, thermal ablation of FNH can give patients a better medical experience and is both economic and practical, with significant advantages over surgical treatment.

In recent years, rehabilitation or enhanced recovery programs for liver surgery have been gradually applied to clinical practice. Relevant clinical studies have shown that compared with traditional standardized treatment, preoperative rehabilitation programs and perioperative steroid administration can effectively reduce overall complications (21, 22). In this study, complications of the ablation group were only compared with those of FNH surgical standardized treatment. In the future, we will conduct in-depth research between the complications of prehabilitation or enhanced recovery program for liver surgery and the thermal ablation complications to further verify the clinical value of thermal ablation.

In conclusion, thermal ablation has less trauma, fewer complications, and is economical and practical. For patients with hepatic FNH ≤5 cm who need active treatment, especially the elderly, children, and those who cannot tolerate surgery, thermal ablation is a safe and effective treatment method worthy of recommendation.

## Data availability statement

The original contributions presented in the study are included in the article/supplementary material. Further inquiries can be directed to the corresponding authors.

## Ethics statement

The studies involving human participants were reviewed and approved by the ethical and scientific review board of Fujian Provincial Hospital. The patients/participants provided their written informed consent to participate in this study.

## Author contributions

All authors listed have made a substantial, direct, and intellectual contribution to the work, and approved it for publication.

## Conflict of interest

The authors declare that the research was conducted in the absence of any commercial or financial relationships that could be construed as a potential conflict of interest.

## Publisher’s note

All claims expressed in this article are solely those of the authors and do not necessarily represent those of their affiliated organizations, or those of the publisher, the editors and the reviewers. Any product that may be evaluated in this article, or claim that may be made by its manufacturer, is not guaranteed or endorsed by the publisher.
